# The subtype‐specific molecular function of *SPDEF* in breast cancer and insights into prognostic significance

**DOI:** 10.1111/jcmm.16760

**Published:** 2021-06-30

**Authors:** Ting Ye, Jingyuan Li, Jia Feng, Jinglan Guo, Xue Wan, Dan Xie, Jinbo Liu

**Affiliations:** ^1^ Department of Laboratory Medicine The Affiliated Hospital of Southwest Medical University Sichuan China

**Keywords:** breast cancer, molecular function, prognostic significance, *SPDEF*, subtype‐specific

## Abstract

Breast cancer (BC) is a molecular diverse disease which becomes the most common malignancy among women worldwide. There are four BC subtypes (Luminal A, Luminal B, HER2‐enriched and Basal‐like) robustly established following gene expression pattern‐based characterization, behave significant differences in terms of their incidence, risk factors, prognosis and therapeutic sensitivity. Thus, there is an urgent need to provide mechanism research, treatment strategies and/or prognosis evaluation based on the patient stratification of BC subtypes. The prostate‐derived ETS factor *SPDEF* was first identified as an activator of prostate specific antigen, and then, the involvements in many aspects of BC have been proposed. However, the subtype‐specific molecular function of *SPDEF* in BC and insights into prognostic significance have not been clearly elucidated. This study demonstrated for the first time that *SPDEF* may play a diversity role in the expression levels, clinicopathologic importance, biological function and prognostic evaluation in BC via bioinformatics and experimental evidence, which mainly depends on different BC subtyping. In summary, our findings would help to better understand the possible mechanisms of various BC subtypes and to find possible candidate genes for prognostic and therapeutic usage.

## 1 INTRODUCTION

Breast cancer is the most common malignancy among women worldwide ^1^ and also a molecular diverse disease, showing different morphologic and biological characteristics and thus different clinical behaviour and treatment response. As to facilitate oncologic decision‐making, the BC classification systems are developed to provide an accurate diagnosis of the disease and prediction of tumour behaviour. Hereinto, four BC subtypes have been robustly established following gene expression patterns based characterization.^2^ These subtypes, including Luminal A, Luminal B, HER2‐enriched and Basal‐like, behave significant differences in terms of their incidence, risk factors, prognosis and therapeutic sensitivity.^3^, ^4^ Therefore, there is an urgent need to provide mechanism research, treatment strategies and prognosis evaluation based on the patient stratification of BC subtypes.

*SPDEF* was first identified as an activator of prostate specific antigen,^5^ which is largely restricted to epithelial tissues including the lung, stomach, colon and hormone‐regulated epithelia such as the prostate, breast and ovary.^6^ In cancer literatures, the role of *SPDEF* in BC is controversial depends on different subtypes, as several studies have demonstrated that high *SPDEF* expression was confirmed to promote Luminal BC differentiation and correlates with poor overall survival in ER+breast cancer patients.^6^, ^7^, ^8^, ^9^ Furthermore, *SPDEF* can also promote proliferation, migration and invasion of SK‐BR‐3 cells through AR‐PDEF pathway ^10^ or SPDEF‐CEACAM6 oncogenic axis.^11^ The set of above observations exhibits a possible oncogenic function of *SPDEF*. Conversely, the down‐regulation of *SPDEF* in invasive basal breast cancer cell lines supports a tumour suppressive role.^12^, ^13^ Therefore, the discrepancies between these findings and those on *SPDEF* as an oncogene and/or a tumour suppressor have not been resolved. Further, the potential mechanisms underlying subtype‐specific functions of *SPDEF* remain largely unknown.

Bioinformatics analysis has been widely applied in cancer research. In the present study, we uncovered the global expression profiles of *SPDEF*, as well as the clinicopathologic and prognostic importance in different BC subtypes through TCGA‐BRCA datasets. Moreover, we verified the protein levels of *SPDEF* with immunohistochemical staining and analysed the relationships between the protein expression of *SPDEF* and clinicopathologic features in BC subtypes. These bioinformatics and clinical findings have added a new dimension to our knowledge about *SPDEF* in addition to its role only as an oncogene or a tumour suppressor in BC. Afterwards, we explored the potential functions and signal pathways of *SPDEF* in BC subtypes using GO, KEGG and hallmark effect gene set analysis, which demonstrated the potential molecular mechanisms of *SPDEF* underlying the oncogenic activity in non‐TNBC (Lumina and HER2+) but tumour suppressor activity in TNBC. And lastly, we conducted the prognostic risk model of *SPDEF*‐related prognosis genes, respectively, in BC subtypes, indicating a highly prognostic performance in survival surveillance. In this study, we innovatively focussed on the *SPDEF* gene in the aspects of the differential expressions, potential functions and prognostic values in multiple BC subtypes via bioinformatics and experimental evidence. The workflow of the study design is presented in Figure S1.

## 2 MATERIALS AND METHODS

### 2.1 *SPDEF* expression analysis in TCGA‐BRCA dataset

Differential expression of *SPDEF* in non‐tumourous breast tissues and different subtypes of BC tissues were obtained from The Cancer Genome Atlas Project (TCGA). The *SPDEF* mRNA levels in different subtypes of BC were evaluated using edgeR software packages.^14^

### 2.2 Validation of cell lines with RT‐qPCR

Cell lines were purchased from the Cell Bank of the Chinese Academy of Sciences and cultured in special medium. RNA was extracted by TRIZOL (Takara) and transcribed into cDNA using PrimeScript RT reagent Kit (Takara). The quantitative real‐time PCR (qPCR) was used to detect the mRNA expression of *SPDEF* in different subtypes of BC cells. The PCR primers were sequenced as follows: 5’‐ GAGCCACCTGAGGAGCCTGAG −3’ (forward) and 5’‐ CTTGAGCACTTCGCCCACCAC −3’ (reverse) for *SPDEF*; 5’‐ CCGGAATCCCTATCTTTAGTCC −3’ (forward) and 5’‐ GCCTTTGTTGCTCTTCCAAAAT‐3’ (reverse) for *TBP*.

### 2.3 Immunohistochemical staining

The paraffin‐embedded tissues were obtained from the Pathology Department of the Affiliated Hospital of Southwest Medical University. And the tissue slides were deparaffinized, rehydrated and stained with the rabbit polyclonal anti‐SPDEF antibody (AB clonal, 1:300) overnight at 4℃. Next, the slides were treated with biotinylated secondary antibody followed by incubation with streptavidin‐HRP. Finally, there were stained using DAB and counterstained with haematoxylin. SPDEF staining was scored based on the multiplier of the positive percentage and staining intensity of the stained area as a result of the total score ranged from 0 to 6. The percentage of SPDEF‐positive stained cells was scored as 0 (0%–25%), 1 (25%–50%) and 2 (>50%). In addition, the intensity of SPDEF expression was scored as 0 no staining (−), 1 weak staining (+), 2 moderate staining (++) and 3 strong staining (+++). A total score of ≥4 indicated positive SPDEF expression.

### 2.4 The clinicopathologic and prognostic analysis of *SPDEF* in BC patients

The association between the *SPDEF* expression and overall survival was performed by Kaplan‐Meier method.^15^ To combine with clinical data of patients, the clinical significance of *SPDEF* expression was figured out. And the best performing threshold is used as a cut‐off.

### 2.5 GO function and KEGG pathway enrichment analysis

Aberrantly expressed genes were filtered using transcription profiles from TCGA‐BRCA database. The correlation coefficients were calculated based on Pearson in order to find the *SPDEF*‐related genes among differentially expressed genes (r > 0.4, *P* < .05). And then, the bioinformatic analysis of the *SPDEF*‐related genes involved GO Enrichment analysis^16^ and KEGG signal transduction pathway enrichment^17^ were performed by R software and Bioconductor packages.^18^

### 2.6 Gene set enrichment analysis

The different subtypes of BC patients were divided into high‐ and low‐expression groups based on the median expression level of *SPDEF* from TCGA‐BRCA database. Hallmark effector gene set of high *SPDEF* expression was annotated by gene set enrichment analysis (GSEA).^19^, ^20^ Hallmark effector gene sets were obtained from the Molecular Signature Database (MsigDB).^21^ The *P*‐value <0.05 and false discovery rate (FDR) <0.25 were used as cut‐off criterion.

### 2.7 Construction of prognostic risk model of BC patients based on *SPDEF*‐related genes

Firstly, univariate Cox regression analysis was performed to identify significant prognostic genes in *SPDEF*‐related genes from TCGA database (*P* < .05). Then, the least absolute shrinkage and selection operator (LASSO) Cox^22^ model was used to identify most critical *SPDEF*‐related prognostic genes. Moreover, risk score model and predictive signature model of prognosis were built by the multivariate Cox regression. According to the median value of the risk score, all patients from TCGA database were divided into the high‐risk group and low‐risk group to perform the evaluation of Kaplan‐Meier (K‐M) survival curves.

### 2.8 Statistical analysis

The expression levels of gene expression levels in between breast cancer and normal breast tissues were statistically compared by Student's t test and Wilcoxon signed rank sum test. Data were analysed by GraphPad Prism 7.0 software and R‐4.0.2 software, which presented as mean ± SEM. Differences were considered statistically significant when *P* < .05.

## 3 RESULTS

### 3.1 The differential expressions of *SPDEF* in multiple subtypes of BC

**FIGURE 1:**
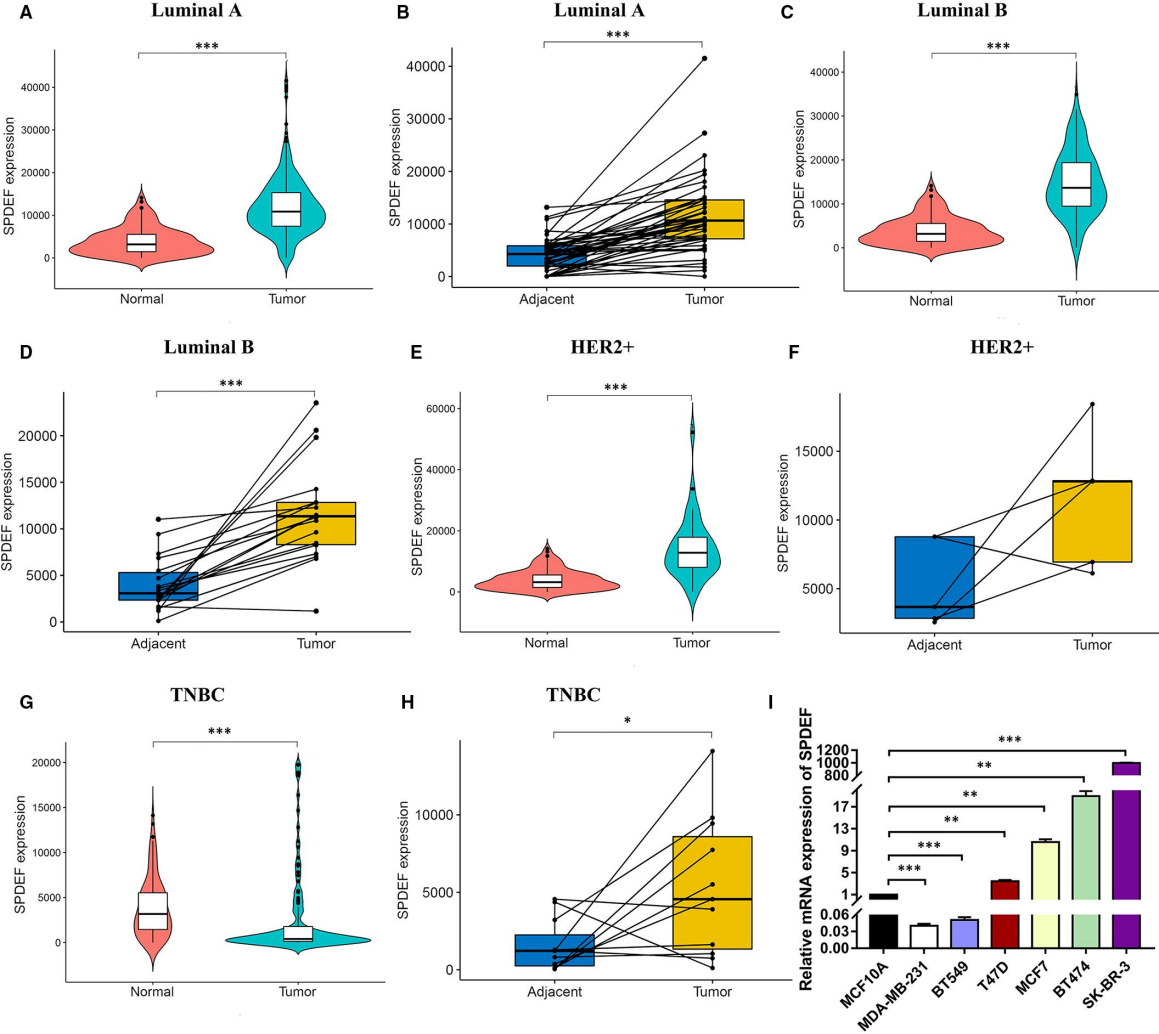
The global *SPDEF* expression profiles in different BC subtypes.(A‐H). *SPDEF* expression level in different subtypes of BC samples compared to normal pericarcinomatous samples. The mRNA levels of *SPDEF* in unmatched BC and matched BC were downloaded from TCGA datasets. (A‐B) Luminal A, (C‐D) Luminal B, (E‐F) HER2+, (G‐H) TNBC. I. mRNA expression of *SPDEF* in different subtypes of BC cells. *: *P* < .05; **: *P* < .01; ***: *P* < .001

We first analysed the mRNA expression of *SPDEF* between BC subtypes and normal (adjacent) breast tissues using TCGA database. *SPDEF* was remarkably overexpressed due to increased mRNA in non‐TNBC (Luminal A, Luminal B and HER2+) compared to normal individuals or adjacent tissues (Figure 1A‐F). Nevertheless, the mRNA expression levels of *SPDEF* in TNBC tissues were dramatically decreased compared with that in the normal or adjacent breast tissues (Figure 1G‐H). To further validate the results of TCGA database, we conducted *SPDEF* expression analyses using the GEPIA2, TIMER website databases and GEO datasets for the expression of *SPDEF* in BC subtypes. Consistently, the *SPDEF* expression is significant up‐regulated in non‐TNBC compared with that in normal tissues, but opposite in TNBC (Figure S2A‐C). Meanwhile, the expression of *SPDEF* in different subtype BC cells (MCF7, T47D, BT474, SK‐BR‐3, MDA‐MB‐231, BT549) and its corresponding group (MCF 10A) was detected by RT‐qPCR. The results showed that non‐TNBC cells (MCF7, T47D, BT474, SK‐BR‐3) had elevated *SPDEF* mRNA, whereas TNBC (MDA‐MB‐231, BT549) cells had decreased expression in comparison with the non‐malignant MCF 10A cells (Figure 1I).

**FIGURE 2:**
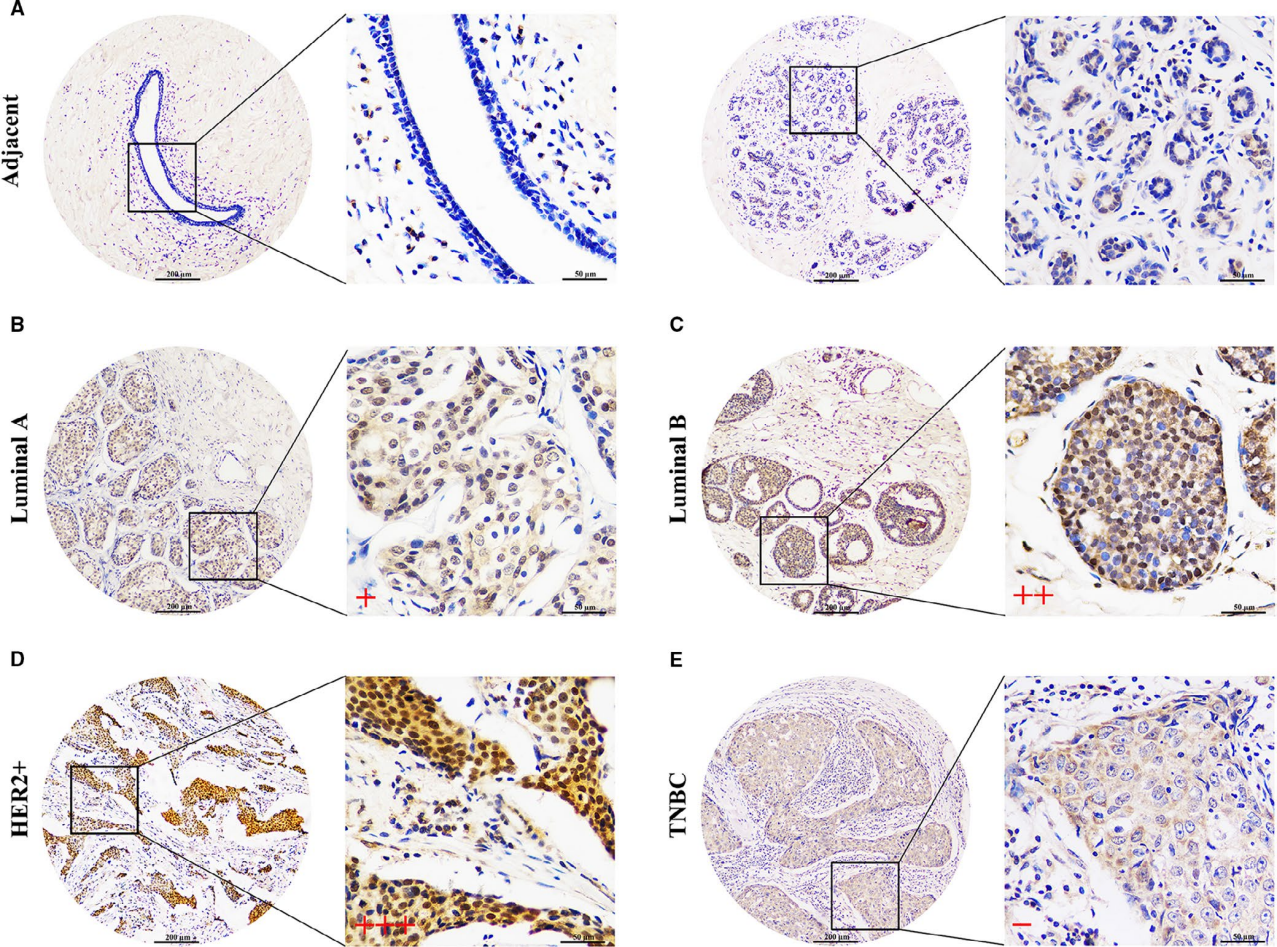
Clinical validation of *SPDEF* expression in multiple BC subtypes. Representative immunohistochemical staining of *SPDEF* in multiple BC subtypes. (A) Adjacent tissue, (B) Luminal A tissue, (C) Luminal B tissue, (D) HER2+ tissue, (E) TNBC tissue

Further, to verify the findings of the bioinformatic analysis, we detected the protein expression of *SPDEF* with immunohistochemical staining. The paraffin‐embedded tissues were collected for *SPDEF* protein analysis, including different subtypes BC cases (Luminal A, Luminal B, HER2+ and TNBC) and their matched adjacent normal tissues. What can be clearly seen in immunohistochemical figures is that the *SPDEF* expression was significantly up‐regulated in Luminal A, Luminal B and HER2+ BC tissues compared with corresponding adjacent normal tissues (Figure 2A‐D). And the positive staining of *SPDEF* was mostly distributed in the nucleus. Rather, there was no significant change in *SPDEF* protein expression in TNBC tissues compared with normal tissues (Figure 2E). Taken together, our data support that *SPDEF* is up‐regulated in the non‐TNBC, but suppressed in TNBC. Besides, the relationships between the protein expression of *SPDEF* and clinicopathologic features in BC subtypes are summarized in Table 1. Over‐expressed protein of *SPDEF* was significantly associated with lymphatic metastasis (*P* = .039) in Luminal A. As for the Luminal B and HER2+, high *SPDEF* expression was positively associated with TNM stage (*P* = .046 in Luminal B, *P* = .023 in HER2+) and lymphoid nodal status (*P* = .019 in Luminal B, *P* = .043 in HER2+). However, no significant difference was found in TNBC.

**TABLE 1:** Relationships between the protein expression of *SPDEF* and clinicopathological parameters in four molecular subtypes of BC based on IHC detection

Variables	*SPDEF* expression in Luminal A			*P*‐value	*SPDEF* expression in Luminal B			*P*‐value	*SPDEF* expression in HER2+			*P*‐value	*SPDEF* expression in TNBC			*P‐*value
	Total (n = 42)	Negative (%) 23 (54.8)	Positive (%) 19 (45.2)		Total (n = 86)	Negative (%) 36 (41.9)	Positive (%) 50 (58.1)		Total (n = 39)	Negative (%) 21 (53.8)	Positive (%) 18 (46.2)		Total (n = 58)	Negative (%) 49 (84.5)	Positive (%) 9 (15.5)	
Age at Surgery																
≤49	18	9 (50.0)	9 (50.0)	0.591	40	17 (42.5)	23(57.5)	0.911	14	9 (64.3)	5 (35.7)	0.504	21	18 (85.7)	3 (14.3)	0.856
>49	24	14 (58.3)	10 (41.7)		46	19 (41.3)	27 (58.7)		25	12 (48.0)	13 (52.0)		37	31 (83.8)	6 (16.2)	
cTNM stage																
I + II	23	14 (60.9)	9 (39.1)	0.382	49	16 (32.7)	33 (67.3)	0.046*	19	14 (73.7)	5 (26.3)	0.023*	41	34 (82.9)	7 (17.1)	0.913
III + IV	19	9 (47.4)	10 (52.6)		37	20 (54.1)	17 (45.9)		20	7 (35.0)	13 (65.0)		17	15 (88.2)	2 (11.8)	
Lymphatic metastasis																
No	16	12 (75.0)	4 (25.0)	0.039*	34	9 (26.5)	25 (73.5)	0.019*	14	11 (78.6)	3 (21.4)	0.043*	31	26 (83.9)	5 (16.1)	0.822
Positive	26	11 (42.3)	15 (57.7)		52	27(51.9)	25 (48.1)		25	10 (40.0)	15 (60.0)		27	23 (85.2)	4 (14.8)	
Distant Metastasis																
M0	37	20 (54.1)	17 (45.9)	0.820	82	34 (41.5)	48 (58.5)	0.856	37	20 (81.1)	17 (18.9)	1.000	56	47 (83.9)	9 (16.1)	1.000
M1	5	3 (60.0)	2 (40.0)		4	2 (50.0)	2 (50.0)		2	1 (50.0)	1 (50.0)		2	2 (100.0)	0 (0.0)	
Ki‐67																
<14%	42	—	—	—	32	15 (46.9)	17 (53.1)	0.468	6	4 (66.7)	2 (33.3)	0.667	5	5 (100.0)	0 (0.0)	1.000
≥14%	0	—	—		54	21 (38.9)	33 (61.1)		33	17 (51.5)	16 (48.5)		53	44 (83.0)	9 (17.0)	

* Bold values indicate *P* < .05.

### 3.2 The clinicopathologic and prognostic importance of *SPDEF* in different BC subtypes

**FIGURE 3:**
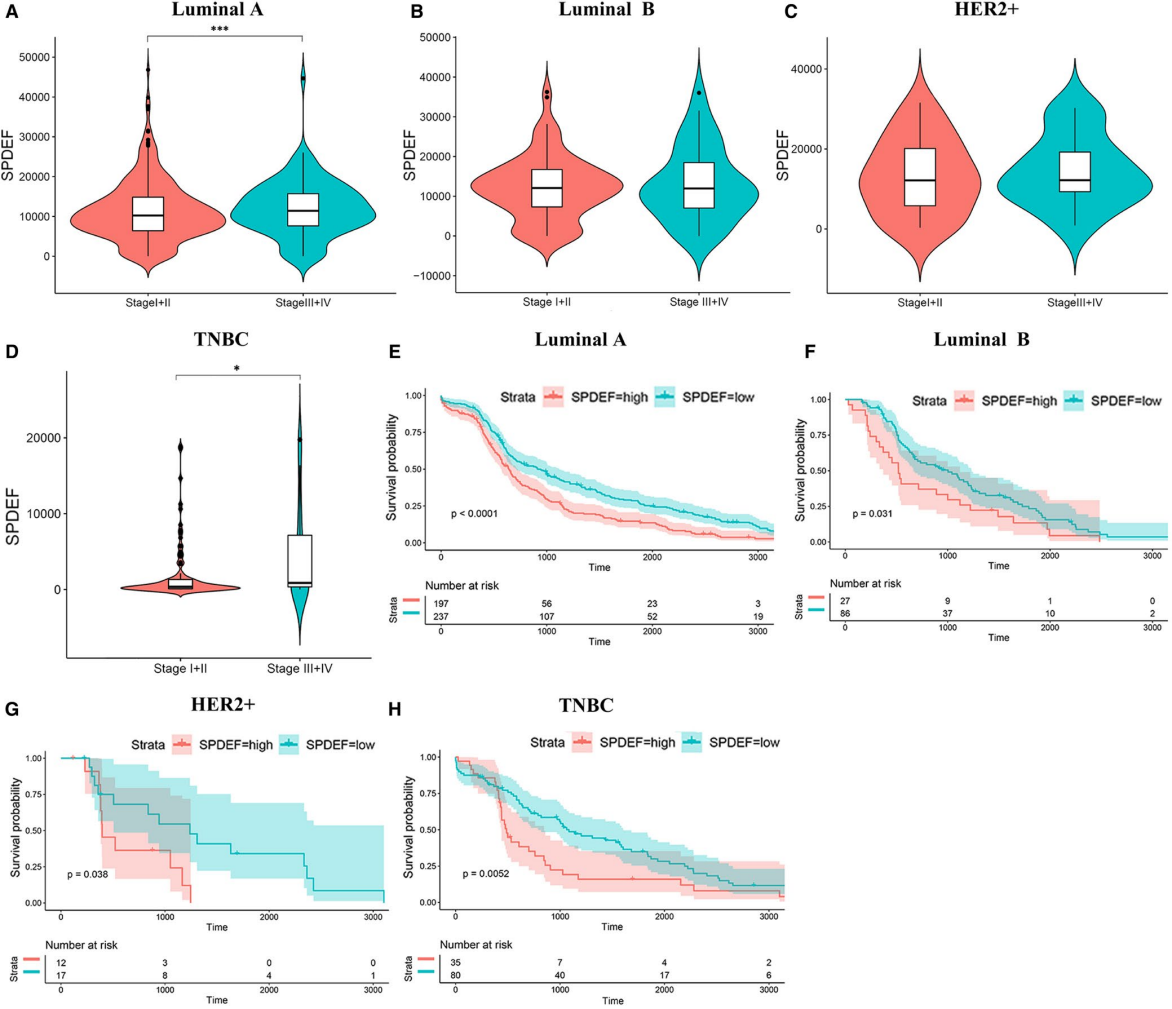
The significance of *SPDEF* in evaluating the clinicopathologic characteristics and prognosis of BC patients across different subtypes.(A‐D). Differential expressions of *SPDEF* in early and late tumour stage according to BC subtypes. (A) Luminal A, (B) Luminal B, (C) HER2+, (D) TNBC. E‐H. Kaplan‐Meier estimates of the overall survival of patients with different BC subtypes according to *SPDEF* levels. (E) Luminal A, (F) Luminal B, (G) HER2+, (H) TNBC. *: *P* < .05; ***: *P* < .001

In addition, we compared the transcription levels of *SPDEF* among groups of different subtype BC patients, according to different clinicopathological characteristics (Figure 3A‐D) (Table 2). It is demonstrated that no significant difference was found in age and distant metastasis status. Notably, in Luminal A, high *SPDEF* expression was positively associated with TNM stage (*P* = .004), lymphoid nodal status (*P* = .023), whereas in Luminal B, high *SPDEF* expression was positively associated with tumour invasion (*P* = .025). As for HER2+, the overexpression of *SPDEF* was positive correlation with lymphoid nodal status (*P* = .032). And it also showed a positive association between *SPDEF* increased mRNA and TNM stage (*P* = .032) in TNBC. Afterwards, we analysed the prognostic value of *SPDEF* expression by examining the relationship between *SPDEF* expression and progression of BC subtyping using TCGA database by Kaplan‐Meier method. Interestingly, high *SPDEF* mRNA levels are correlated with faster disease progression and lower rate of overall survival (OS) in all subtypes BC (Figure 3E‐H). Furthermore, BC patients with a low *SPDEF* expression exhibited a better distance metastasis‐free survival (DMFS) compared with patients with a high *SPDEF* expression by the Kaplan‐Meier Plotter website analysis (Figure S1D‐G). Thus, high *SPDEF* expression predicts poor prognosis.

**TABLE 2:** The association of *SPDEF* mRNA expression and clinicopathological parameters in BC subtypes based on TCGA database

Variables	*SPDEF* mRNA expression of luminal A			*P*‐value	*SPDEF* mRNA expression of luminal B			*P*‐value	*SPDEF* mRNA expression of HER2+			*P*‐value	*SPDEF* mRNA expression of TNBC			*P*‐value
	Total(n = 442)	Low (n = 221)	High (n = 221)		Total(n = 126)	Low (n = 63)	High (n = 63)		Total(n = 37)	Low (n = 19)	High (n = 18)		Total(n = 115)	Low (n = 58)	High (n = 57)	
Age at surgery																
<51	121	60	61	0.915	36	16	20	0.430	10	5	5	0.920	45	25	20	0.379
≥51	321	161	160		90	47	43		27	14	13		70	33	37	
cTNM Stage								0.588								
Ⅰ + Ⅱ	327	177	150	0.004*	90	44	46		25	15	10	0.129	92	51	41	0.032*
Ⅲ + Ⅳ	110	42	68		35	19	16		12	4	8		23	7	16	
X	5	2	3		1		1									
Tumour invasion																
T1 + T2	365	188	177	0.200	107	58	49	0.025*	32	16	16	0.677	99	49	50	0.616
T3 + T4	76	33	43		19	5	14		5	3	2		16	9	7	
X	1		1													
Lymphoid nodal status																
‐	193	109	84	0.023*	52	24	28	0.423	10	8	2	0.032*	74	41	33	0.152
+	244	111	133		73	39	34		25	10	15		41	17	24	
X	5	1	4		1		1		2	1	1					
Distant metastasis status																
M0	377	195	182	0.467	101	49	52	0.299	36	19	17	0.298	98	52	46	0.137
M1	6	4	2		4	3	1		1	0	1		2	0	2	
MX	59	22	37		21	11	10		10				15	6	9	

* Bold values indicate *P* < .05.

### 3.3 The Gene Ontology functions enrichment analysis of *SPDEF*‐related genes in various BC subtyping

**FIGURE 4:**
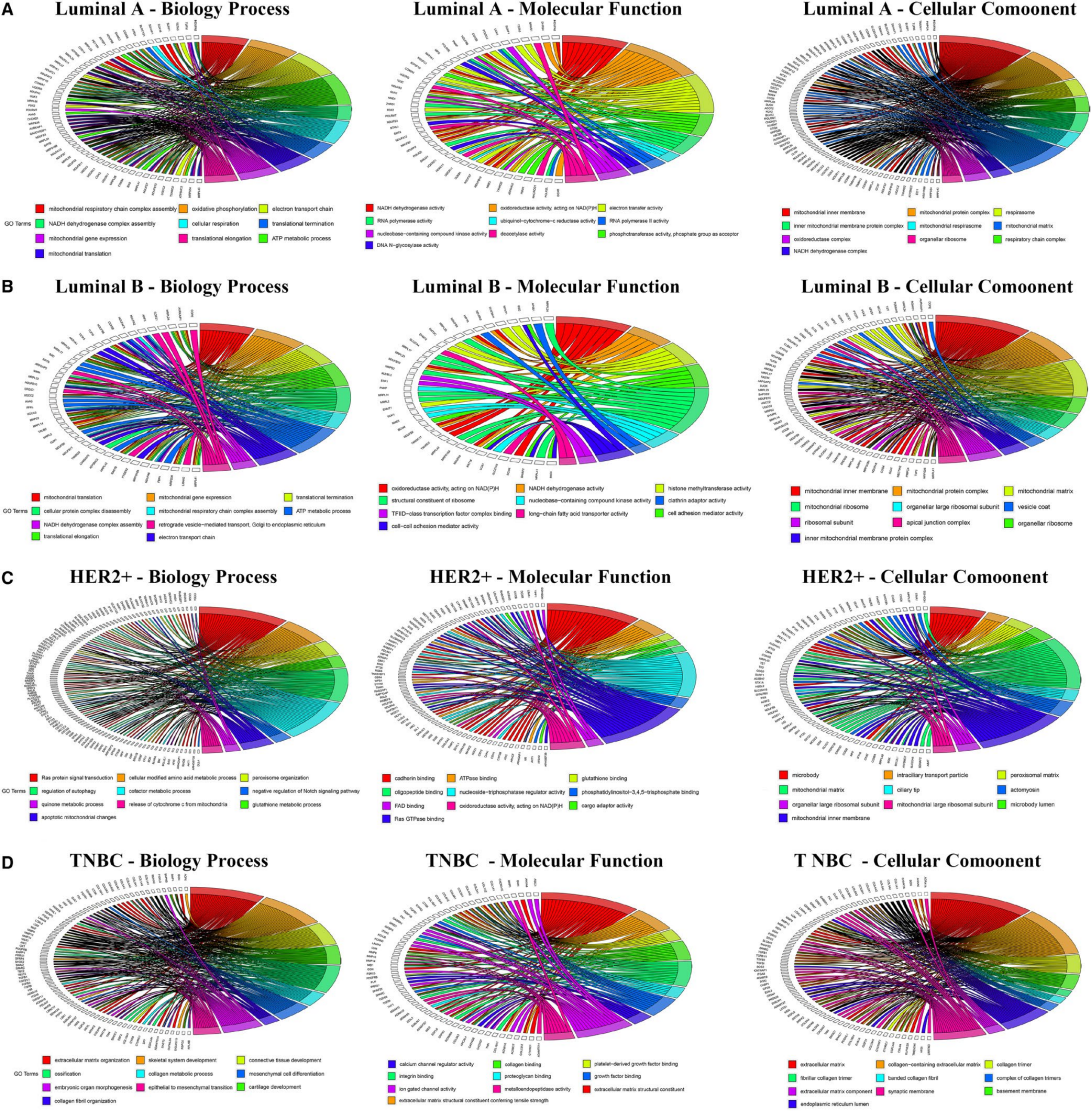
GO annotation enrichment analysis of *SPDEF*‐related genes in various BC subtyping. Main biological processes, molecular functions and cell components related to *SPDEF* were identified by *SPDEF*‐related genes. (A) Luminal A, (B) Luminal B, (C) HER2+, (D) TNBC

To better understand the gene‐enrichment and functional annotation analyses of *SPDEF*, we implemented GO enrichment to discovery the functions in which the *SPDEF* participated in BC subtyping, with a threshold of *P* < .05. The overview schematic of analysis results is displayed in Figure 4 and Table S1. The functions of the gene *SPDEF* were enriched analysis according to the GO terms of the biological process (BP), cellular component (CC) and molecular function (MF). As the top 10 of GO enrichment illustrated in Luminal A, the GO term of ‘mitochondrial respiratory chain complex assembly’ (GO: 0033108) was the most significant enrichment for BP category (*P* < .001). In the MF category, ‘NADH dehydrogenase activity’ (GO: 0003954) was the highest enrichment term (*P* < .001). And the GO term of ‘mitochondrial inner membrane’ (GO: 0005743) was the most important of the CC category (*P* < .001). In Luminal B, the GO term of ‘mitochondrial translational’ (GO: 0032543), ‘oxidoreductase activity, acting on NAD(P)H’ (GO: 0016651), ‘mitochondrial inner membrane’ (GO: 0005743) were the most prominent enrichment for BP (*P* < .001), MF (*P* < .001) and CC (*P* < .001) category, respectively. And for HER2+ BC, the highest enrichment term was the ‘Ras protein signal transduction’ (GO: 0007265) in BP (*P* < .001), ‘cadherin binding’ (GO: 0045296) in MF (*P* < .001), ‘microbody’ (GO: 0042579) in CC (*P* < .001).

Unlike the enrichment functions of non‐triple negative BC, as the top 10 of GO enrichment illustrated in TNBC, the GO term of ‘extracellular matrix organization’ (GO: 0030198) was the highest enrichment term for BP category (*P* < .001). For MF category, ‘extracellular matrix structural constituent’ (GO: 0005201) was the most significant enrichment. And the GO term of ‘extracellular matrix’ (GO: 0031012) was the most valuable of CC category (*P* < .001).

### 3.4 Enrichment analysis identifies the *SPDEF*‐related signalling pathway in multiple BC subtypes

**FIGURE 5:**
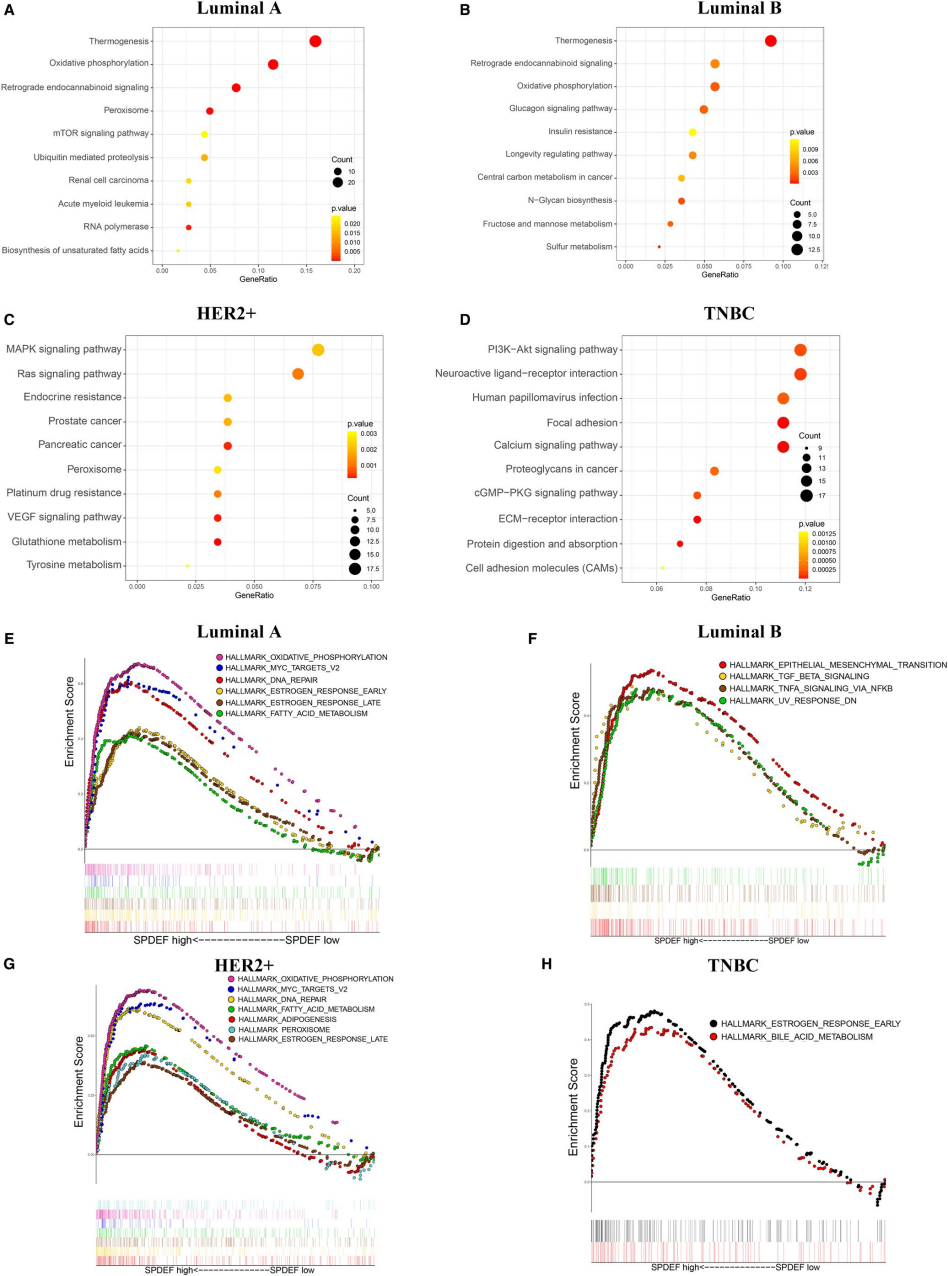
KEGG pathways and Hallmark gene set enrichment analysis associated with *SPDEF* in subtypes of BC. (A‐D). The enriched pathways among the *SPDEF* high expression group were identified in different BC subtypes by KEGG analysis. (A) Luminal A, (B) Luminal B, (C) HER2+, (D) TNBC. E‐H. GSEA profiles depicted significant hallmark gene sets associated with *SPDEF* expression in different BC subtypes. (E) Luminal A, (F) Luminal B, (G) HER2+, (H) TNBC

The deeper molecular functions of *SPDEF* were obtained via KEGG signalling pathway gene sets and evaluating hallmark effect gene sets. All the most valuable enriched pathway of each category were presented, respectively (*P* < .05). Hereinto, the top five KEGG pathway enrichment analysis was shown to be significantly associated with thermogenesis, oxidative phosphorylation, retrograde endocannabinoid signalling, peroxisome and mTOR signalling pathway in Luminal A; thermogenesis, retrograde endocannabinoid signalling, oxidative phosphorylation, glucagon signalling pathway and insulin resistance in Luminal B; MAPK signalling pathway, Ras signalling pathway, endocrine resistance, prostate cancer and pancreatic cancer in HER2+ (Figure 5A‐C). Notably, KEGG results in TNBC indicated enrichment mainly for PI3K‐Akt signalling pathway, neuroactive ligand‐receptor interaction, human papillomavirus infection, focal adhesion and calcium signalling pathway (Figure 5D).

Besides, the predefined hallmark effect gene sets of different BC subtyping were differentially enriched with the high *SPDEF* expression phenotype (Figure 5E‐H). In Luminal A, *SPDEF*‐related signalling pathways included DNA repair, oestrogen response early/late, fatty acid metabolism, MYC targets V2 and oxidative phosphorylation, whereas in Luminal B, *SPDEF*‐related signalling pathways included epithelial‐mesenchymal transition (EMT), TGF‐β signalling, TNFA signalling via NFKB and UV response DN. For HER2+, the pathways enriched in adipogenesis, DNA repair, oestrogen response late, fatty acid metabolism, MYC targets V2, oxidative phosphorylation and peroxisome are similar to those of Luminal A. And TNBC‐related signalling pathways include bile acid metabolism and oestrogen response early. This suggests that *SPDEF* may contribute to different biological functions in the development of various BC subtypes.

### 3.5 Construction of the prognostic risk model of *SPDEF*‐related prognosis genes in subtypes of BC

**FIGURE 6:**
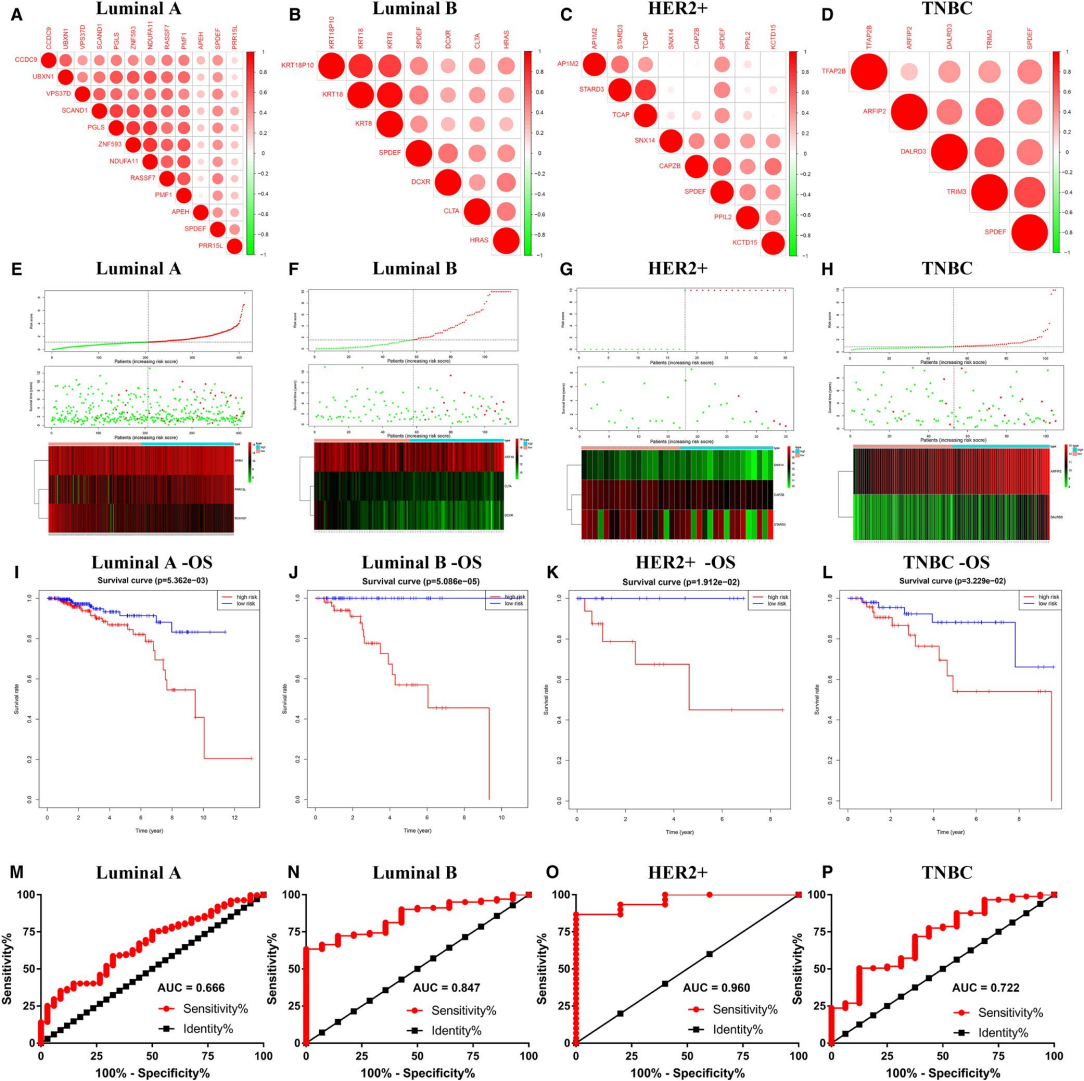
Prognostic risk score model analysis of *SPDEF*‐related prognostic genes with different BC subtypes(A‐D). Correlation analysis between *SPDEF* and co‐expressed prognostic genes in subclasses of BC. (A) Luminal A, (B) Luminal B, (C) HER2+, (D) TNBC. E‐H. The risk score distribution, survival status of patients and heatmap of gene expression pattern in different BC subtypes. (E) Luminal A, (F) Luminal B, (G) HER2+, (H) TNBC. I‐L. The Kaplan‐Meier plot for OS of patients in the different risk groups of BC subtypes. (I) Luminal A, (J) Luminal B, (K) HER2+, (L) TNBC. M‐P. The ROC curves for the prognostic value of the risk score in different BC subtypes. (M) Luminal A, (N) Luminal B, (O) HER2+, (P) TNBC

To further investigate the clinical prognostic effect of *SPDEF* in multiple BC subtyping, we firstly performed to identify prognostic genes of *SPDEF*‐correlated from TCGA database by univariate Cox regression analysis. And then, we obtained 11 genes (CCDC9, UBXN1, VPS37D, SCAND1, PGLS, ZNF593, NDUFA11, RASSF7, PMF1, APEH, PRR15L) in Luminal A, 6 genes (KRT18P10, KRT18, KRT8, DCXR, CLTA, HRAS) in Luminal B, 7 genes (AP1M2, STARD3, TCAP, SNX14, CAPZB, PPIL2, KCTD15) in HER2+ and 4 genes (TFAP2B, ARFIP2, DALRD3, TRIM3) in TNBC, respectively (Figure 6A‐D).

Based on the results of *SPDEF*‐related prognostic genes analysis, we developed a prognostic index (PI) to stratify different subtypes of BC patients into two groups (high and low risk) and constructed a predictive model to identify the performance of PI in predicting the clinical outcome of BC subtype patients. The formula of PI is as follows: (0.00014 * expression value of APEH) + (0.00019 * expression value of PRR15L) + (−0.00050 * expression value of SCAND1) in Luminal A, (0.00004 * expression value of KRT18) + (−0.00044 * expression value of CLTA) + (−0.00051 * expression value of DCXR) in Luminal B, (0.00150 * expression value of STARD3) + (−0.01141 * expression value of CAPZB) + (−0.03763 * expression value of SNX14) in HER2+, (0.00025 * expression value of ARFIP2) + (0.00071 * expression value of DALRD3) in TNBC. The distribution of risk scores, survival status of each subject and heatmap of gene expression pattern are shown in Figure 6E‐H. And the higher risk score showed a shorter survival time for patients and vice versa (Figure 6I‐L). The area under the curve (AUC) of the receiver operating characteristic (ROC) was 0.666 in Luminal A, 0.847 in Luminal B, 0.960 in HER2+, 0.722 in TNBC (Figure 6M‐P), indicating a high prognostic performance of the *SPDEF*‐related prognostic genes in survival surveillance.

## 4 DISCUSSION

Breast cancer is a clinically and biologically heterogeneous disease; thus, research based on BC subtypes is critical to achieve better clinical outcomes.^23^ In cancer literature, the role of *SPDEF*, known as the prostate‐derived ETS factor, that functions in BC is widely reported. Prior to the present study, we have summarized *SPDEF* as the double agent involving in expression profiles, the regulator mechanism in BC progression, as well as the role in diagnosis, treatment and prognosis of BC with literature review.^24^ However, the specific roles of *SPDEF* in various subtypes of BC have not been systematically evaluated and established. This study demonstrated for the first time that *SPDEF* may play a diversity role in the expression levels, clinicopathologic importance, biological function and prognostic evaluation in BC via bioinformatics and experimental evidence, which mainly depends on different BC subtyping.

We made the following novel findings that had not been previously reported:

First, the oncogene function of *SPDEF* overexpression in non‐TNBC (Luminal A, Luminal B, HER2+) and the tumour suppressor function of *SPDEF* down‐regulation in TNBC have been uncovered by bioinformatics analysis (Figure 1A‐H). Subsequently, the overabundance of *SPDEF* in non‐TNBC (Luminal A, Luminal B, HER2+) relative to TNBC has been verified by the transcription level detection in variety BC cells (Figure 1I) and the protein analysis in paraffin‐embedded tissues of BC subtypes (Figure 2A‐E). Moreover, high‐protein level of *SPDEF* was positively associated with lymphatic metastasis in Luminal A, with TNM stage and lymphoid nodal status in Luminal B and HER2+, but no significant difference in TNBC (Table 1). Thus, this set of observations suggests the differential expression of *SPDEF* which allowed the characteristics of the pro‐ and anti‐oncogenic activities in various BC subtype. Future in‐depth mechanism governing the regulation of *SPDEF* in BC subtypes will contribute to gain insight into the BC biology and also add a new dimension to the new treatment targets rather than treating BC as a single entity.

Second, the clinicopathologic and prognostic values of *SPDEF* in various BC subtypes have been explored and established. Here, we demonstrated that high *SPDEF* mRNA levels were positively correlated with faster disease progression in Luminal A, and TNBC (Figure 3A‐D). In‐depth analysis of BC from TCGA databases shows the poor overall survival of *SPDEF* high expression (Figure 3E‐H), which merits further investigation to establish whether it is a new prognostic marker for the four BC subtypes. In addition, high transcript level of *SPDEF* was positively related to TNM stage, lymphoid nodal status in Luminal A, with tumour invasion in Luminal B, with lymphoid nodal status in HER2+, and even with TNM stage in TNBC (Table 2). These observations indicated that high levels of *SPDEF* expression promote the BC progression which has distinctive characteristics of subtypes, respectively, laying the foundation for future mechanism research.

Third, this study was the first attempt to predict that *SPDEF* participated in tumorigenesis and progression of BC subtypes by GO analysis, which was involved in the aspects of biological process (BP), cellular component (CC) and molecular function (MF) (Table S1). For Luminal BC, the results demonstrated that the enrichment is mainly concentrated on the mitochondrial respiratory and translational (Figure 4A‐B). Consistent with our findings, recent literature sheds light on the contribution of mitochondrial respiration in BC tumorigenesis ^25^ and metastasis,^26^ but lack subtype exploration. And the mitochondrial translational was also demonstrated to be involved in the targeted therapy for leukaemia,^27^, ^28^ which deserves further study in the field of BC. In addition, the bifunctional RasGAP tumour suppressor has been proved to be concomitantly suppressed in aggressive luminal B tumours and drive metastasis by activating RAS signal transduction.^29^ Herein, we proposed for the first time that Ras protein signal transduction was closely related to HER2+ BC by GO analysis (Figure 4C), which is worth further exploring through experimental evidences. Meanwhile, related to our analysis of TNBC (Figure 4D), extracellular matrix organization has been reported to participate in the regulation process that GREM1 promotes the invasion and metastasis of ER‐negative breast.^30^ Hence, as for TNBC, in‐depth mechanistic characteristics of cancerogenesis and development referring to extracellular matrix merits further investigation.

Fourth, we have innovatively predicted the potential signalling pathways associated with *SPDEF* in BC subsets via KEGG and hallmark effect gene set analysis. Above mentioned pathways, the most enrichment pathways were referring to the thermogenesis and oxidative phosphorylation pathways in Luminal A group, the thermogenesis and EMT pathways in Luminal B, the MAPK and oxidative phosphorylation pathways in HER2+, the PI3K‐Akt and oestrogen response early pathways in TNBC (Figure 5). A recent report indicated the disruption of hypoxia‐inducible fatty acid‐binding protein 7 induces beige fat‐like differentiation and thermogenesis in breast cancer cells, in which the rise in temperature of cancer cells may impact on patients’ outcomes.^31^ EMT pathways were also proved to be responsible for metastases and therapy resistance in Luminal B type BC.^32^, ^33^ Additionally, seldom literature showed the MAPK pathways were involved in the metastasis of HER2+ type BC cells,^34^ and mitochondrial oxidative phosphorylation was correlated with the promotion of chemotherapy‐resistant BC stem cells.^35^ And the evidence from a phase 1 trial verified the targeting of the PI3K/AKT/mTOR pathway for the treatment of mesenchymal TNBC.^36^ Noteworthy, there is another study regarding the value of ERβ‐targeted therapies for the treatment of TNBC patients,^37^ which was closely correlated and consistent with the oestrogen response early pathways enriched in TNBC in our results. Taken together, *SPDEF* may carry out its regulation functions in such BC subtypes through participation in above signalling pathways. This need to be clarified by further researches.

Fifth, the prognostic risk model of *SPDEF*‐related prognosis genes in subtypes of BC has been constructed for the first time, indicating a high prognostic performance in survival surveillance. The *SPDEF*‐based prognostic index could be an important tool for distinguishing among various subtyping BC patients based on potential discrete outcomes (Figure 6A‐H). Furthermore, this prognostic index can effectively and accurately stratify different subtypes of BC patients, which is vital for monitoring the survival of subtype‐specific patients (Figure 6L). And the ROC curves revealed a high predictive value of the risk model (Figure 6M‐P). Notably, there were two advantages of using the *SPDEF*‐related prognosis genes to construct the prognostic risk model in different subtypes of breast cancer. On the one hand, the influence of confounding factors in the analysis process could be avoided to ensure the inclusion of *SPDEF*‐related prognostic genes significantly associated with the survival outcome. On the other hand, the optimum point of the performance parameters was determined, which improved the discrimination ability of the prognostic risk model. In summary, our findings provide new insights that can guide a more detailed assessment of BC patients in subsequent clinical trials.

In conclusion, the study we presented here indicated that specific expressions and molecular functions of *SPDEF* might lead to the occurrence and development of multiple BC subtypes. Further, high expression of *SPDEF* shows the poor OS and subtype‐specific risk model of *SPDEF*‐related prognosis genes indicated a high prognostic performance in survival surveillance in various BC. Overall, our findings would help to better understand the possible mechanisms of various BC subtypes and to find possible candidate genes for prognostic and therapeutic usage.

## CONFLICT OF INTEREST

The authors declare no conflict of interest.

## AUTHOR CONTRIBUTIONS

**Ting Ye:** Conceptualization (lead); Data curation (lead); Formal analysis (lead); Funding acquisition (lead); Project administration (lead); Validation (lead); Visualization (lead); Writing‐original draft (lead). **Jingyuan Li:** Data curation (lead); Formal analysis (lead); Validation (lead). **Jia Feng:** Data curation (equal); Formal analysis (equal). **Jinglan Guo:** Data curation (equal); Formal analysis (equal). **Xue Wan:** Data curation (equal); Formal analysis (equal). **Dan** **Xie:** Data curation (equal); Formal analysis (equal). **Jinbo Liu:** Conceptualization (lead); Funding acquisition (lead); Project administration (lead); Writing‐review & editing (lead).

## Supporting information

Fig S1

Fig S2

Table S1

Supplementary Material

## Data Availability

All data utilized in this study are included in this article, and all data supporting the findings of this study are available on reasonable request from the corresponding authors.
