# Supplementary figures and images for: The subtype‐specific molecular function of SPDEF in breast cancer and insights into prognostic significance
Source: J Cell Mol Med. 2021 Jun 30;25(15):7307–20. doi: 10.1111/jcmm.16760 (PMC8335683; doi:10.1111/jcmm.16760)

**Supplementary Figure S1**


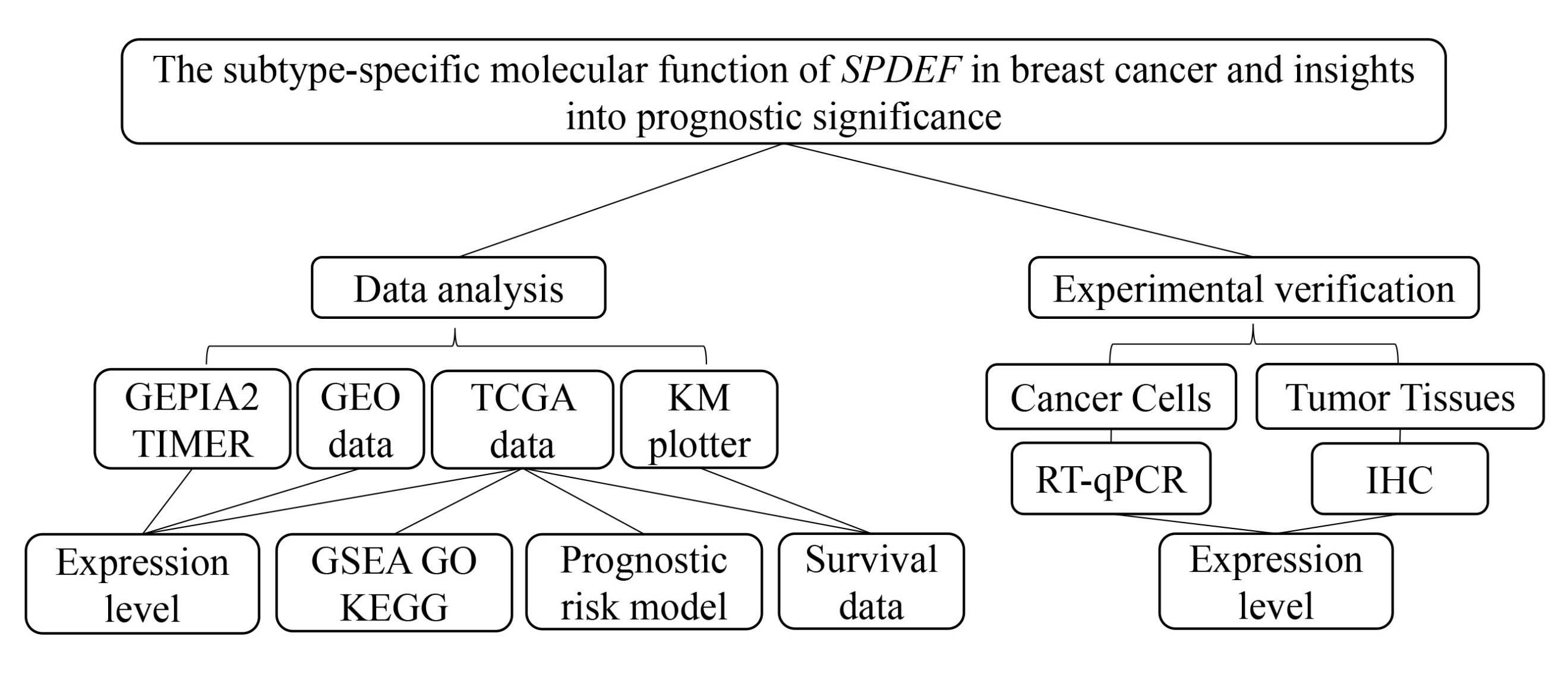


**Figure S1.** Flow chart of the study design.

Supplement: Supplementary file 1 — Fig S1 [file JCMM-25-7307-s001.docx]
