# Supplementary material for: The subtype‐specific molecular function of SPDEF in breast cancer and insights into prognostic significance
Source: J Cell Mol Med. 2021 Jun 30;25(15):7307–20. doi: 10.1111/jcmm.16760 (PMC8335683; doi:10.1111/jcmm.16760)
Supplement: Supplementary file 2 — Fig S2 [file JCMM-25-7307-s003.docx]

**Supplementary Figure S2**


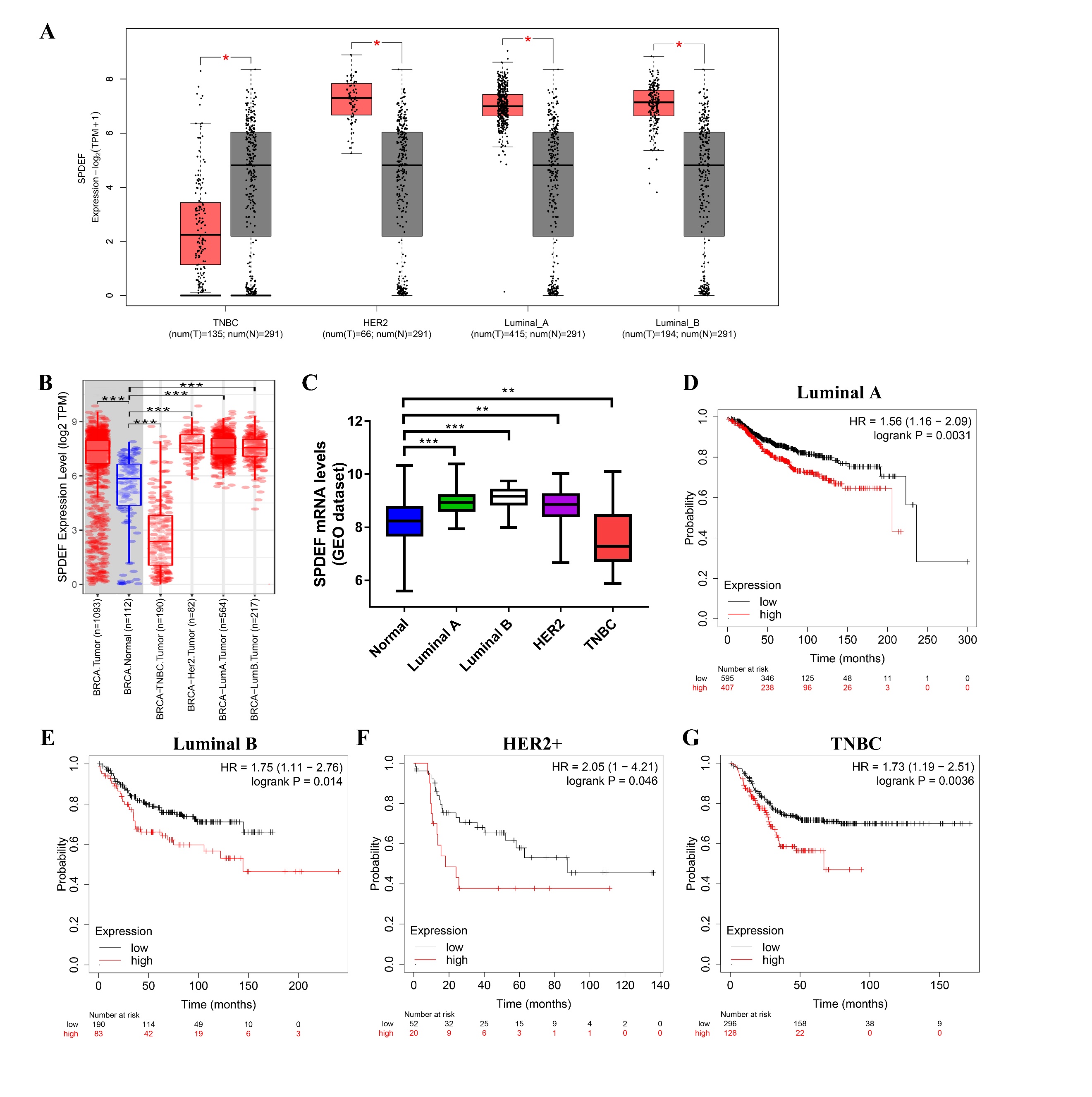


**Figure S2.** The differential expression and prognostic value of *SPDEF* in different BC subtypes. (A) The expression level of *SPDEF* in different subtypes of BC tissues and normal tissues in the GEPIA database. (B) The expression level of *SPDEF* in different BC subtypes of tumor tissues and normal tissues in TIMER database. (C) GEO data showing the expression profiles of *SPDEF* in normal tissue vs different BC subtypes. (D-G) Kaplan Meier-plotter analysis of the association between *SPDEF* expressions and DMFS in different BC subtypes. (D) Luminal A, (E) Luminal B, (F) HER2+, (G) TNBC.
