# Supplementary material for: The subtype‐specific molecular function of SPDEF in breast cancer and insights into prognostic significance
Source: J Cell Mol Med. 2021 Jun 30;25(15):7307–20. doi: 10.1111/jcmm.16760 (PMC8335683; doi:10.1111/jcmm.16760)
Supplement: Supplementary file 3 — Table S1 [file JCMM-25-7307-s002.docx]

**Table S1 The GO function enrichment analysis of *SPDEF*-related genes in different subtypes of BC**

| GO ID | Term | Ontology | Count | *P*-value |
| --- | --- | --- | --- | --- |
| GO:0033108 | mitochondrial respiratory chain complex assembly | BP | 22 | 3.53E-16 |
| GO:0006119 | oxidative phosphorylation | BP | 24 | 3.79E-14 |
| GO:0022900 | electron transport chain | BP | 24 | 9.91E-12 |
| GO:0046034 | ATP metabolic process | BP | 29 | 1.28E-10 |
| GO:0010257 | NADH dehydrogenase complex assembly | BP | 14 | 1.76E-10 |
| GO:0045333 | cellular respiration | BP | 22 | 9.09E-10 |
| GO:0006415 | translational termination | BP | 16 | 2.18E-09 |
| GO:0032543 | mitochondrial translation | BP | 18 | 2.33E-09 |
| GO:0140053 | mitochondrial gene expression | BP | 19 | 7.37E-09 |
| GO:0006414 | translational elongation | BP | 16 | 7.96E-08 |
| GO:0003954 | NADH dehydrogenase activity | MF | 10 | 1.88E-07 |
| GO:0016651 | oxidoreductase activity, acting on NAD(P)H | MF | 13 | 3.31E-06 |
| GO:0009055 | electron transfer activity | MF | 12 | 3.42E-05 |
| GO:0016776 | phosphotransferase activity, phosphate group as acceptor | MF | 7 | 5.86E-04 |
| GO:0097747 | RNA polymerase activity | MF | 6 | 9.30E-04 |
| GO:0008121 | ubiquinol-cytochrome-c reductase activity | MF | 3 | 1.73E-03 |
| GO:0001055 | RNA polymerase II activity | MF | 3 | 2.33E-03 |
| GO:0019104 | DNA N-glycosylase activity | MF | 3 | 4.85E-03 |
| GO:0019205 | nucleobase-containing compound kinase activity | MF | 5 | 5.04E-03 |
| GO:0019213 | deacetylase activity | MF | 5 | 5.04E-03 |
| GO:0005743 | mitochondrial inner membrane | CC | 56 | 3.03E-24 |
| GO:0098798 | mitochondrial protein complex | CC | 39 | 7.11E-21 |
| GO:0070469 | respirasome | CC | 21 | 8.95E-15 |
| GO:0098803 | respiratory chain complex | CC | 19 | 5.09E-14 |
| GO:0098800 | inner mitochondrial membrane protein complex | CC | 23 | 5.50E-14 |
| GO:0005746 | mitochondrial respirasome | CC | 19 | 1.00E-13 |
| GO:0005759 | mitochondrial matrix | CC | 41 | 2.14E-13 |
| GO:0030964 | NADH dehydrogenase complex | CC | 13 | 6.68E-11 |
| GO:1990204 | oxidoreductase complex | CC | 16 | 7.36E-09 |
| GO:0000313 | organellar ribosome | CC | 13 | 8.91E-08 |

**Luminal A**

| GO ID | Term | Ontology | Count | *P*-value |
| --- | --- | --- | --- | --- |
| GO:0032543 | mitochondrial translation | BP | 15 | 2.16E-08 |
| GO:0140053 | mitochondrial gene expression | BP | 15 | 2.47E-07 |
| GO:0006415 | translational termination | BP | 12 | 3.73E-07 |
| GO:0006414 | translational elongation | BP | 13 | 8.46E-07 |
| GO:0043624 | cellular protein complex disassembly | BP | 14 | 4.10E-05 |
| GO:0033108 | mitochondrial respiratory chain complex assembly | BP | 9 | 5.84E-05 |
| GO:0046034 | ATP metabolic process | BP | 16 | 1.35E-04 |
| GO:0022900 | electron transport chain | BP | 12 | 1.44E-04 |
| GO:0010257 | NADH dehydrogenase complex assembly | BP | 7 | 1.48E-04 |
| GO:0006890 | retrograde vesicle-mediated transport, Golgi to endoplasmic reticulum | BP | 8 | 1.59E-04 |
| GO:0016651 | oxidoreductase activity, acting on NAD(P)H | MF | 8 | 1.24E-03 |
| GO:0003954 | NADH dehydrogenase activity | MF | 5 | 2.02E-03 |
| GO:0042054 | histone methyltransferase activity | MF | 5 | 5.18E-03 |
| GO:0098631 | cell adhesion mediator activity | MF | 5 | 6.00E-03 |
| GO:0003735 | structural constituent of ribosome | MF | 10 | 6.81E-03 |
| GO:0019205 | nucleobase-containing compound kinase activity | MF | 4 | 1.07E-02 |
| GO:0035615 | clathrin adaptor activity | MF | 2 | 1.56E-02 |
| GO:0098632 | cell-cell adhesion mediator activity | MF | 4 | 1.66E-02 |
| GO:0001094 | TFIID-class transcription factor complex binding | MF | 2 | 1.89E-02 |
| GO:0005324 | long-chain fatty acid transporter activity | MF | 2 | 1.89E-02 |
| GO:0005743 | mitochondrial inner membrane | CC | 33 | 1.56E-11 |
| GO:0098798 | mitochondrial protein complex | CC | 23 | 2.01E-10 |
| GO:0005759 | mitochondrial matrix | CC | 26 | 2.53E-07 |
| GO:0000313 | organellar ribosome | CC | 11 | 3.43E-07 |
| GO:0005761 | mitochondrial ribosome | CC | 11 | 3.43E-07 |
| GO:0000315 | organellar large ribosomal subunit | CC | 9 | 5.95E-07 |
| GO:0030120 | vesicle coat | CC | 7 | 6.60E-05 |
| GO:0098800 | inner mitochondrial membrane protein complex | CC | 10 | 1.34E-04 |
| GO:0044391 | ribosomal subunit | CC | 12 | 1.36E-04 |
| GO:0043296 | apical junction complex | CC | 10 | 2.16E-04 |

**Luminal B**

| GO ID | Term | Ontology | Count | *P*-value |
| --- | --- | --- | --- | --- |
| GO:0007265 | Ras protein signal transduction | BP | 32 | 2.23E-06 |
| GO:0006575 | cellular modified amino acid metabolic process | BP | 18 | 8.18E-06 |
| GO:0007031 | peroxisome organization | BP | 11 | 1.91E-05 |
| GO:0006749 | glutathione metabolic process | BP | 9 | 2.78E-05 |
| GO:0010506 | regulation of autophagy | BP | 24 | 2.80E-05 |
| GO:0051186 | cofactor metabolic process | BP | 29 | 4.34E-05 |
| GO:0045746 | negative regulation of Notch signaling pathway | BP | 7 | 2.01E-04 |
| GO:0008637 | apoptotic mitochondrial changes | BP | 12 | 2.35E-04 |
| GO:1901661 | quinone metabolic process | BP | 6 | 2.59E-04 |
| GO:0001836 | release of cytochrome c from mitochondria | BP | 8 | 2.65E-04 |
| GO:0045296 | cadherin binding | MF | 22 | 6.79E-04 |
| GO:0051117 | ATPase binding | MF | 8 | 3.30E-03 |
| GO:0043295 | glutathione binding | MF | 3 | 4.05E-03 |
| GO:0140312 | cargo adaptor activity | MF | 3 | 4.05E-03 |
| GO:1900750 | oligopeptide binding | MF | 3 | 5.27E-03 |
| GO:0060589 | nucleoside-triphosphatase regulator activity | MF | 20 | 5.44E-03 |
| GO:0005547 | phosphatidylinositol-3,4,5-trisphosphate binding | MF | 5 | 6.75E-03 |
| GO:0017016 | Ras GTPase binding | MF | 22 | 1.48E-02 |
| GO:0071949 | FAD binding | MF | 4 | 1.65E-02 |
| GO:0016651 | oxidoreductase activity, acting on NAD(P)H | MF | 8 | 1.81E-02 |
| GO:0042579 | microbody | CC | 14 | 2.84E-05 |
| GO:0030990 | intraciliary transport particle | CC | 6 | 9.09E-05 |
| GO:0005782 | peroxisomal matrix | CC | 7 | 5.63E-04 |
| GO:0031907 | microbody lumen | CC | 7 | 5.63E-04 |
| GO:0005759 | mitochondrial matrix | CC | 26 | 9.74E-04 |
| GO:0097542 | ciliary tip | CC | 6 | 1.62E-03 |
| GO:0042641 | actomyosin | CC | 8 | 1.79E-03 |
| GO:0005743 | mitochondrial inner membrane | CC | 24 | 4.66E-03 |
| GO:0000315 | organellar large ribosomal subunit | CC | 6 | 5.44E-03 |
| GO:0005762 | mitochondrial large ribosomal subunit | CC | 6 | 5.44E-03 |

**HER2+**

| GO ID | Term | Ontology | Count | *P*-value |
| --- | --- | --- | --- | --- |
| GO:0030198 | extracellular matrix organization | BP | 36 | 2.09E-14 |
| GO:0001501 | skeletal system development | BP | 34 | 1.23E-08 |
| GO:0061448 | connective tissue development | BP | 22 | 5.36E-07 |
| GO:0051216 | cartilage development | BP | 19 | 6.00E-07 |
| GO:0001503 | ossification | BP | 27 | 1.41E-06 |
| GO:0032963 | collagen metabolic process | BP | 14 | 2.13E-06 |
| GO:0048762 | mesenchymal cell differentiation | BP | 18 | 9.06E-06 |
| GO:0030199 | collagen fibril organization | BP | 9 | 3.21E-05 |
| GO:0048562 | embryonic organ morphogenesis | BP | 20 | 5.01E-05 |
| GO:0001837 | epithelial to mesenchymal transition | BP | 14 | 6.76E-05 |
| GO:0005201 | extracellular matrix structural constituent | MF | 25 | 8.99E-14 |
| GO:0030020 | extracellular matrix structural constituent conferring tensile strength | MF | 12 | 1.64E-09 |
| GO:0048407 | platelet-derived growth factor binding | MF | 6 | 2.70E-06 |
| GO:0005518 | collagen binding | MF | 10 | 1.74E-05 |
| GO:0005178 | integrin binding | MF | 13 | 1.74E-05 |
| GO:0043394 | proteoglycan binding | MF | 6 | 3.84E-03 |
| GO:0019838 | growth factor binding | MF | 10 | 5.33E-03 |
| GO:0005246 | calcium channel regulator activity | MF | 6 | 5.33E-03 |
| GO:0022839 | ion gated channel activity | MF | 15 | 7.55E-03 |
| GO:0004222 | metalloendopeptidase activity | MF | 8 | 1.02E-02 |
| GO:0031012 | extracellular matrix | CC | 43 | 2.50E-16 |
| GO:0062023 | collagen-containing extracellular matrix | CC | 39 | 7.10E-16 |
| GO:0005581 | collagen trimer | CC | 16 | 2.51E-10 |
| GO:0005604 | basement membrane | CC | 13 | 6.70E-07 |
| GO:0005583 | fibrillar collagen trimer | CC | 6 | 7.02E-07 |
| GO:0098643 | banded collagen fibril | CC | 6 | 7.02E-07 |
| GO:0098644 | complex of collagen trimers | CC | 7 | 7.02E-07 |
| GO:0005788 | endoplasmic reticulum lumen | CC | 22 | 1.41E-06 |
| GO:0044420 | extracellular matrix component | CC | 8 | 6.07E-05 |
| GO:0097060 | synaptic membrane | CC | 22 | 8.83E-05 |

**TNBC**
