# Supplementary material for: The subtype‐specific molecular function of SPDEF in breast cancer and insights into prognostic significance
Source: J Cell Mol Med. 2021 Jun 30;25(15):7307–20. doi: 10.1111/jcmm.16760 (PMC8335683; doi:10.1111/jcmm.16760)
Supplement: Supplementary file 4 — Supplementary Material [file JCMM-25-7307-s004.docx]

**Supplementary Materials and methods**

***SPDEF* expression analysis in GEPIA2 Dataset**

The online database Gene Expression Profiling Interactive Analysis 2 (GEPIA2) (http://gepia2.cancer-pku.cn/#index) is a newly interactive web server for analyzing the RNA sequencing expression data from the TCGA and the Genotype-Tissue Expression (GTEx) projects, using a standard processing pipeline. The GEPIA2 was used to further confirm the expressions of *SPDEF* in BC subtypes.

**TIMER database analysis**

TIMER (https://cistrome.shinyapps.io/timer/) is a database designed for analyzing gene expression in multiple cancers. The TIMER database was employed to assess differential expression of *SPDEF* in different BC subtypes.

**GEO microarray** **dataset analysis**

To verify the differential expression of *SPDEF* in different BC subtypes, we searched mRNA expression profiling datasets in the Gene Expression Omnibus (GEO) (<https://www.ncbi.nlm.nih.gov/geo/>). We screened 2 expression profiles, GSE93601 and GSE93601. After quality control and batch effect elimination of the two datasets, the combination of the two expression profiles was served as the dataset for gene expression analysis. We used the R package of “limma” to identify the differential expression analysis.

**Kaplan-Meier Plotter Website Analysis**

Kaplan Meier-plotter (http://kmplot.com/analysis/) is a commonly used website tool for assessing the effect of genes on survival based on EGA (European Genome-phenome Archive), TCGA and GEO database. The distant metastasis-free survival (DMFS) curves of *SPDEF* were generated by the Kaplan-Meier plots.
